# Heart work after errors: Behavioral adjustment following error commission involves cardiac effort

**DOI:** 10.3758/s13415-018-0576-6

**Published:** 2018-02-20

**Authors:** Iris M. Spruit, Tom F. Wilderjans, Henk van Steenbergen

**Affiliations:** 1 Cognitive Psychology Unit, Institute of Psychology, Leiden University, Wassenaarseweg, 52 2333 AK Leiden, The Netherlands; 2 Research Group of Methodology and Statistics, Institute of Psychology, Leiden University, Leiden, The Netherlands; 3 Research Group of Quantitative Psychology and Individual Differences, Faculty of Psychology and Educational Sciences, KU Leuven, Leuven, Belgium; 4 Leiden Institute for Brain and Cognition, Leiden, The Netherlands

**Keywords:** Errors, Effort, Cognitive control, Orienting response, Cardiac measures, Heart rate, Pre-ejection period, RZ interval

## Abstract

Posterror slowing (PES) is the observation that people respond slower on trials subsequent to error commissions than on trials subsequent to correct responses. Different accounts have been proposed to explain PES. On the one hand, it has been suggested that PES arises from an adaptive increase in cognitive control following error commission, thereby making people more cautious after making an error. On the other hand, PES has been attributed to an orienting response, indicating that attention is shifted toward the error. In the present study we tested these accounts by investigating the effects of error commission in both flanker and switch tasks on two task-evoked cardiac measures: the interbeat interval—that is, the interval between two consecutive R peaks—and the RZ interval—that is, the interval between the R peak and the Z point—as measured using electro- and impedance cardiography, respectively. These measures allowed us to measure cardiac deceleration (autonomic orienting) and cardiac effort mobilization, respectively. Our results revealed a shorter RZ interval during posterror trials, indicating increased effort mobilization following errors. In addition, we replicated earlier studies that have shown cardiac slowing during error trials. However, multilevel analyses showed that only the posterror decrease in RZ interval predicted posterror reaction times, whereas there was no positive relationship between error-related cardiac deceleration and posterror reaction times. Our results suggest that PES is related to increased cardiac effort, supporting a cognitive-control account of PES.

**Electronic supplementary material:**

The online version of this article (10.3758/s13415-018-0576-6) contains supplementary material, which is available to authorized users.

The ability to adapt behavior in response to challenges, such as making errors, is a critical aspect of goal-directed behavior. One key observation in cognitive-control tasks is that people respond slower on trials following error commissions than on trials following correct responses, a phenomenon called *posterror slowing* (PES; Laming, 1979; Rabbitt, 1966). PES has proven to be a robust finding observed in various tasks, including the flanker (Cavanagh, Cohen, & Allen, 2009), Stroop (Gehring & Fencsik, 2001), Simon (Ridderinkhof, 2002), categorization (Jentzsch & Dudschig, 2009), and task-switching (Themanson, Hillman, & Curtin, 2006) paradigms. Although PES was described as early as the 1960s (Rabbitt, 1966), there is still debate regarding its underlying mechanisms (Danielmeier & Ullsperger, 2011).

According to the influential conflict-monitoring theory, PES occurs due to adjustments in cognitive control, implemented to improve behavior following errors (Botvinick, Braver, Barch, Carter, & Cohen, 2001; Yeung, Botvinick, & Cohen, 2004). More specifically, this account holds that PES occurs due to response conflict associated with the error, which leads to improved cognitive control by increasing the response threshold for the subsequent trial (Botvinick et al., 2001). Therefore, responses are slower and more often correct following error trials. However, a more recent account has proposed that PES occurs due to an orienting response to the error (Notebaert et al., 2009; cf. Dudschig & Jentzsch, 2009). According to this orienting account, attention is shifted toward the error because these are typically rare events. This orienting response (OR) is thought to involve a time-consuming orientation to the error followed by a reorientation to the task, which produces the slower response in trials following an error. The involuntary switch in attention toward rare events can be measured at the physiological level (Pavlov, 1927; Sokolov, 1963) and accompanies a set of responses including pupil dilation, increased skin conductance (Sokolov, 1963), and heart rate deceleration (Graham & Clifton, 1966; Lynn, 1966). Consistent with the orienting account of PES, many studies have observed these physiological reactions in response to errors, which include pupil dilation (Braem, Coenen, Bombeke, van Bochove, & Notebaert, 2015; Critchley, Tang, Glaser, Butterworth, & Dolan, 2005; Murphy, van Moort, & Nieuwenhuis, 2016; Rondeel, van Steenbergen, Holland, & van Knippenberg, 2015; Wessel, Danielmeier, & Ullsperger, 2011), skin conductance (Crone, Somsen, van Beek, & van der Molen, 2004; Hajcak, McDonald, & Simons, 2003), and heart rate deceleration (Crone et al., 2003; Danev & Winter, 1971; Fiehler, Ullsperger, Grigutsch, & von Cramon, 2004; Hajcak et al., 2003; Somsen, van der Molen, Jennings, & van Beek, 2000; van der Veen, van der Molen, & Jennings, 2000; Wessel et al., 2011). Furthermore, some of these physiological reactions to errors have been linked to PES, such as pupil dilation (Murphy et al., 2016) and the skin conductance response (Hajcak et al., 2003), although heart rate deceleration (Hajcak et al., 2003) has not. Thus, findings on the relationship between the OR and PES are still mixed and inconclusive.

Alternatively, and in line with a cognitive-control account of PES, it is possible that PES is related to an increase in effort. This notion is supported by research that has shown that facial electromyographic activity in the corrugator supercilii increases after error commission and predicts PES (Elkins-Brown, Saunders, He, & Inzlicht, 2017; Elkins-Brown, Saunders, & Inzlicht, 2016; Lindström, Mattsson-Mårn, Golkar, & Olsson, 2013). Indeed, this physiological measure has been used as an index of effort (de Morree & Marcora, 2010; van Boxtel & Jessurun, 1993),—although it could also reflect the aversiveness of errors (Inzlicht, Bartholow, & Hirsh, 2015; Koban & Pourtois, 2014; Saunders & Jentzsch, 2012) and/or its associated response conflict (Botvinick, 2007; Dreisbach & Fischer, 2012; van Steenbergen, 2015). Thus, it is possible that errors elicit effort mobilization, which aligns well with a cognitive-control account of PES (Botvinick et al., 2001; Yeung et al., 2004) and with recent work that has suggested that the concepts of cognitive control and effort are strongly related (Shenhav et al., 2017; see also Hasher & Zacks, 1979; Kahneman, 1973; Kool & Botvinick, 2014; Mulder, 1986).

In the present study, we investigated the mechanism responsible for PES by testing the roles of the OR and effort, respectively using two different cardiac measures. The OR was measured by cardiac deceleration, as can be demonstrated by an increase in the length of the interval between heart beats (interbeat interval, IBI) following an error (Crone et al., 2003; Danev & Winter, 1971; Fiehler et al., 2004; Hajcak et al., 2003; Somsen et al., 2000; van der Veen et al., 2000; Wessel et al., 2011). On the other hand, effort was measured using the RZ interval. The RZ interval (RZI) is the time interval between the R peak, which is obtained from electrocardiography (ECG) measurements and reflects ventricular depolarization, and the Z point (dZ/dt_max_), which is obtained from impedance cardiography (ICG) measurements and reflects the peak in aortic blood flow. This RZI is a single-trial proxy of the pre-ejection period (PEP), which reflects the sympathetic effect on the contractility of the heart (Obrist, 1981). Numerous studies have shown that PEP becomes shorter with increased task difficulty, rendering it a reliable index of cognitive effort (Gendolla & Richter, 2010; Gendolla, Wright, & Richter, 2011; Richter, Friedrich, & Gendolla, 2008). However, because PEP cannot be accurately measured at the single-trial level, we have recently developed a method to analyze task-evoked differences in the RZI (Kuipers et al., 2017), which closely relates to PEP (Lozano et al., 2007), thereby allowing us to measure cardiac effort at the single-trial level. Using this method, we tested whether PES indeed involves increased effort mobilization, an effect that might be observed immediately following an error (cf. Murphy et al., 2016) and/or at the subsequent trial (Botvinick et al., 2001).

To sum up, following the orienting account of PES, we expected cardiac deceleration in error as compared to correct trials, which might reflect an adaptive OR that predicts PES. However, it is also possible that PES is the result of an increase in effort. If this is true, the RZI, the cardiac index of effort mobilization, should decrease after error relative to correct trials and should predict PES. These hypotheses were tested in a study that measured PES in a flanker and a switch task, while cardiac measures were obtained.

## Material and method

### Participants

The data from the flanker and switch task described here were collected as part of a larger study investigating the effects of working posture (sitting versus alternating between sitting and standing) on cognitive performance (data to be published elsewhere). The study was approved by the ethics committee of the Leiden University Psychology department. Participants provided written informed consent prior to participation and received course credits or participation in a lottery to potentially win a coupon worth €25 upon completion. A total of 60 students completed the two sessions of this study. Inclusion criteria were: age between 18 and 30 years, fluent in Dutch, no dreadlocks or braids, no use of medication (excluding contraceptives and allergy medication), no physical limitations that makes sitting or standing painful or impossible, and no psychiatric illnesses or head injury (excluding minor concussion). After data collection 13 participants were excluded from further analyses. Eight were excluded because of errors in data collection that caused substantial missing ECG and ICG data (at least one block), and three participants were excluded because of poor ECG and/or ICG signals. Furthermore, two participants were excluded because their data included extreme outliers (more than three interquartile ranges above or below the 25th/75th percentile) on one or more behavioral scores in one or both tasks. Thus, a total of 47 participants were included in the reported analyses (39 female, 36 right handed, and with a mean age of 19.98 years). This sample size is sufficient to be sensitive to medium to large effect sizes reported earlier in related studies (e.g., Hajcak et al., 2003; Murphy et al., 2016) with a power of 80%.

### Overview

The study consisted of two sessions, each lasting approximately 2 h. In one session the participants sat down while performing the tasks. In the other session the participants alternated per block between sitting and standing while performing the tasks. To keep our findings compatible to typical lab studies, only the data collected during the sitting sessions were analyzed. The participants performed three tasks, a modified version of the flanker task (Eriksen & Eriksen, 1974; Lorist, Boksem, & Ridderinkhof, 2005), a number switch task (Rondeel et al., 2015), and a working memory task (two-back task). The tasks were performed in six blocks and each block took approximately 18 min. The order of the tasks in each block stayed constant for each participant, but varied across participants. Analyses were limited to the flanker task and switch task. These specific tasks were chosen because they require speeded responses and have been shown to elicit error-related behavioral and physiological responses (Lorist et al., 2005; Rondeel et al., 2015). During task performance, electrocardiography (ECG) and impedance cardiography (ICG) were used to obtain cardiac measures. Electroencephalography (EEG) data were also collected, but these are not considered in the present analyses.

### Tasks

E-Prime 2.0 was used to present the tasks. Participants were instructed to respond as fast and accurate as possible while performing the tasks. For all the tasks responses were made with the right and left index fingers by pressing the “q” and “p” buttons on the keyboard. The stimuli were presented in white on a black background. During the tasks the response keys were displayed on the left and right lower corner of the screen as a reminder in Courier New font, size 10. During the practice block, auditory feedback was provided after each trial indicating whether the participant pressed the right button, the wrong button, or no button. No feedback was provided during the test blocks, because although error awareness facilitates error-related behavioral and cardiac effects (Klein et al., 2007; Nieuwenhuis, Ridderinkhof, Blom, Band, & Kok, 2001; Wessel et al., 2011), it is typically high in speeded forced manual choice tasks such as these (Ullsperger, Harsay, Wessel, & Ridderinkhof, 2010; Ullsperger & von Cramon, 2006).

For all tasks an accuracy of 85% during the practice block was required before proceeding to the test blocks. Furthermore, a mean reaction time (RT) of 500 ms or below was required for the flanker task and a mean RT of 1,200 ms or below was required for the switch task.

In the flanker task (modified version of task described by Lorist et al., 2005), trials consisted of a cue (150 ms), a fixation cross (850 ms), the stimulus (max 1,000 ms or until a response was made), and a fixation cross. The duration of this last fixation cross depended on RT such that the stimulus reaction time plus the duration of the fixation cross would last for a jittered interval between 1,900 and 2,100 ms. Thus, the total trial duration was jittered between 2,900 and 3,100 ms. The stimuli were congruent (SSS or HHH) 50% of the trials and incongruent (SHS or HSH) the other 50% of the trials. Participants had to respond to the middle (target) letter, indicating whether it was an S or H, with their right and left index fingers, counterbalanced across participants. The letters were printed in green or red; flankers had the same color as the target letter when the stimuli were congruent, whereas flankers had a different color than the target letter when the stimuli were incongruent. A cue preceded stimulus presentation. This cue either concerned the response hand (right or left) or the color of the target letter (green or red). The cue interfered with the correct response in 20% of the trials and facilitated the correct response in the remaining 80% of the trials. The stimuli and cues were presented in bold Palatino Linotype font, sizes 10 and 40, respectively. In total, participants performed 720 trials of the flanker task.

We used a version of the switch task that has been shown to elicit reliable physiological responses to errors (Rondeel et al., 2015). Trials consisted of a stimulus for a maximum of 3,000 ms, or until a response was made, and a fixation cross. The duration of the fixation cross depended on RT such that the total trial duration was jittered between 3,900 and 4,100 ms. The stimuli consisted of the numbers 1 through 9, which were presented in random order. The color of the number could be either yellow or blue. Depending on the color of the number (counterbalanced between participants), participants had to indicate whether the number was odd or even (odd–even task), or whether the number was >5 or ≤5 (size task). The color of the number stayed constant for two trials in a row, and then changed. Thus, trials on which a task was repeated alternated with trials on which the task switched. The stimuli were presented in bold Palatino Linotype font, size 40. In total, participants performed 540 trials of the switch task.

### Cardiac acquisition

To acquire IBI and RZI data, electrocardiography (ECG) and impedance cardiography (ICG) data were obtained with a Biopac MP150 system (Biopac Systems Inc., Goleta, CA, USA). The ECG and ICG data together with event markers to indicate stimulus onset were saved using AcqKnowledge software (Biopac Systems Inc., Goleta, CA, USA). Both ECG and ICG signals were sampled at 1000 Hz. For ECG measurement three Ag/AgCl spot electrodes were used. The first electrode was placed approximately 4 cm below the right clavicle, the second was placed on the lower left abdomen, and the third ground electrode on the lower right abdomen. For ICG measurement eight Ag/AgCl spot electrodes were used. Two were placed on the right side and two on the left side of the neck on the carotid artery. The pairs were positioned five cm apart from each other. The other four were placed in two pairs on each side of the rib cage, in line with the middle of the shoulder, five cm apart from each other. The ICG signal produced measures of basal impedance (Z0) and rate of change in impedance (dZ/dt). The dZ/dt signal together with the ECG signal were used to obtain the RZI data. For details regarding the use of the RZI, see Kuipers et al. (2017). In short, the RZI is the time interval between the R peak in the ECG data, which reflects ventricular depolarization, and the Z (dZ/dtmax) point (Lozano et al., 2007) in the ICG data, which reflects the peak in aortic blood flow. This interval is used as a measure of cardiac effort instead of the more well-known pre-ejection period (PEP). PEP is the time interval ranging from the onset of electromechanical systole to the onset of left-ventricular ejection (Sherwood et al., 1990), but since these points are highly susceptible to noise and distortion, it is not possible to track trial-by-trial changes in PEP. The RZI closely approximates PEP (Lozano et al., 2007) and therefore can be used to investigate cardiac effort at the single-trial level (Kuipers et al., 2017).

### Cardiac preprocessing

The method described by Kuipers et al. (2017) was used to process the ECG and dZ/dt signals in order to obtain heart rate (IBI) and RZI data. Preprocessing was performed in MATLAB release 2012b (The MathWorks, Inc., Natick, MA, USA). The ECG signal was low-pass filtered at 50 Hz to remove high frequency noise and high-pass filtered at 2 Hz to remove low-frequency trends. Zero-phase forward and reverse digital filters were employed in both procedures. R peaks were then automatically detected and IBIs, defined as the time between two consecutive R peaks, were calculated. The filtered ECG signal overlaid with the automatically detected R peaks and IBIs was further inspected by trained persons and any incorrectly detected R peaks and IBIs were removed (proportion accepted R peaks; range = 96.11–99.83%, mean = 99.29%). Because the dZ/dt signals were smooth and did not require detrending, the signals were not further filtered. The dZ/dt_max_ points were automatically identified as the highest peak in the dZ/dt signal in the 300 ms following each R peak. The RZI was calculated as the time interval in milliseconds between each R peak and the corresponding dZ/dt_max_ point (Z). The dZ/dt signal overlaid with the automatically detected dZ/dt_max_ points and RZIs was manually inspected in the same way as the ECG signal (proportion accepted dZ/dt_max_ points given accepted R peaks: range = 95.82%–99.91%, mean = 98.88). Using linear interpolation the IBI and RZI time series were transformed into continuous signals at a sampling rate of 1000 Hz. This interpolation was performed to be able to average the signals across trials. Thus, unlike the method that isolates and analyzes specific IBIs around error commissions, which also allows for investigating cardiac cycle effects (e.g., Lacey & Lacey, 1974; van der Molen, Somsen, & Orlebeke, 1983), we used continuous IBI and RZI time series that allowed us to investigate effects at the level of milliseconds rather than at the level of a few discrete IBIs.

### Behavioral analyses

We first assessed whether participants showed the congruency effect in the flanker task by using paired *t* tests to compare RTs and accuracy on incongruent versus congruent trials. In a similar way, the switch effect in the switch task was assessed by comparing RTs and accuracy on switch versus repetition trials.

For all the following analyses (including the cardiac and multilevel analyses), error and correct triplets were isolated from the data. An error triplet (cEc) consisted of a pre-error trial, an error trial, and a posterror trial. On average, 27.02 error triplets per participant were isolated in the flanker task (*SD* = 11.07, range = 6–51), and 16.72 in the switch task (*SD* = 8.20, range = 3–37). The error trials included only incongruent trials for the flanker task, and switch trials for the switch task. The pre-error and posterror trials consisted only of correct responses; in the flanker task these trials included both congruent and incongruent trials (see Table 1), whereas in the switch task they included only switch trials, because switch and repetition trials alternated. Note that error triplets did not require a correct response on the trials preceding the pre-error trial, so a small portion of the pre-error trials were actually posterror trials. A correct triplet (cCc) consisted of a precorrect trial, a correct trial, and a postcorrect trial. Parallel to the error triplets, the correct trials only consisted of incongruent trials for the flanker task and switch trials for the switch task, and the same characteristics that applied to the pre- and posterror trials applied to the pre- and postcorrect trials.

**Table 1:** Overview of behavioral data

Measure	Flanker			Switch		
	Mean	*SE*	95% CI	Mean	*SE*	95% CI
Correct reaction time (ms)	516.44	7.35	[501.64, 531.24]	866.97	28.77	[809.06, 924.88]
Error reaction time (ms)	449.51	8.11	[433.19, 465.82]	843.98	33.84	[775.88, 912.09]
Pre-error reaction time (ms)	479.30	8.52	[462.15, 496.45]	781.31	27.87	[725.22, 837.40]
Posterror reaction time (ms)	500.07	9.55	[480.84, 519.30]	818.09	29.88	[757.94, 878.24]
Posterror slowing (ms)	20.76	4.72	[11.26, 30.26]	36.78	15.78	[5.02, 68.54]
Postcorrect accuracy (%)	94.00	0.004	[93.1, 94.9]	93.15	0.005	[92.1, 94.2]
Posterror accuracy (%)	95.17	0.009	[93.3, 97.1]	94.91	0.008	[93.3, 96.5]
Posterror accuracy increase (%)	1.17	0.008	[– 0.51, 2.85]	1.77	0.007	[0.27, 3.26]
Pre-error incongruent/switch trials (%)	46.05	1.48	[43.08, 49.03]	0.00	0.00	[0.00, 0.00]
Posterror incongruent/switch trials (%)	45.99	1.33	[43.31, 48.67]	0.00	0.00	[0.00, 0.00]

We assessed whether there was a difference in RTs on error trials (taken from error triplets) versus correct trials (taken from correct triplets) by performing a 2 (Task: flanker or switch) × 2 (Trial: error or correct) repeated measures analysis of variance (ANOVA). Partial eta-squared is reported as the measure of effect size.

**Fig. 1:**
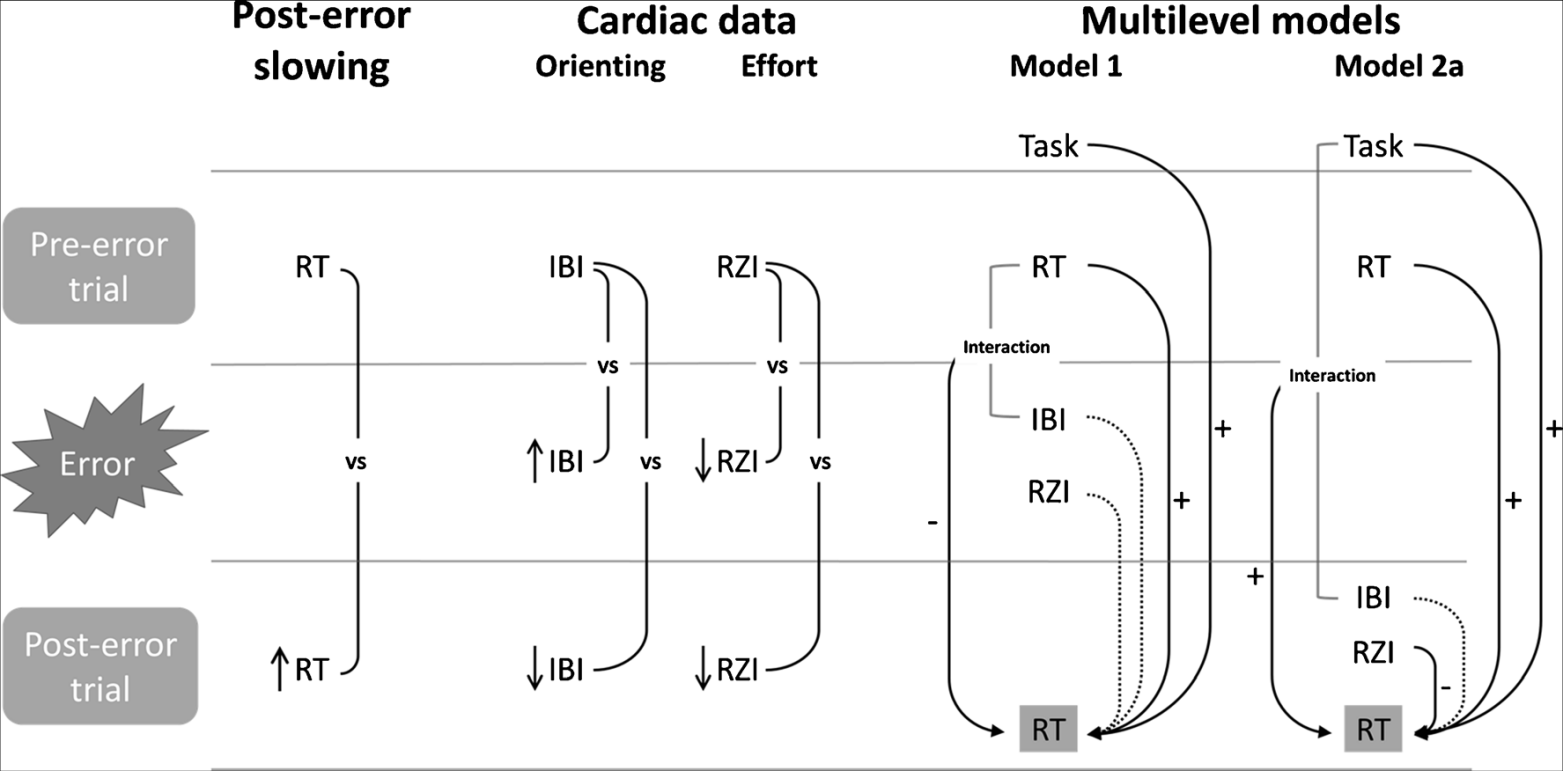
Overview of the main results. (Left) Posterror slowing, as evidenced by increased reaction times (RTs) on posterror as compared to pre-error trials. (Middle) Main comparisons of cardiac analyses on the interbeat interval (IBI) and RZ interval (RZI) waveforms. Note that all the IBI/RZIs in this panel actually reflect the difference between the IBI/RZI on (pre/post)error trials and the IBI/RZI on (pre/post)correct trials. Errors led to an orienting response (increased IBI, and as a result of that, decreased RZI; see the main text) and more cardiac effort in the posterror trial (decreased IBI and decreased RZI). (Right) Summary of the multilevel models: Significant effects are highlighted with + and –. The results showed that the orienting response (Model 1) did not predict posterror RTs, whereas cardiac effort as measured by RZI (Model 2a) did predict posterror RTs. Note that the design of Model 2b was identical to that of Model 2a, except that Model 2b used correct triplets instead of error triplets, and more significant interaction effects were observed in Model 2b.

Next, to assess posterror slowing (PES), we compared the RTs on trials directly following errors with the RTs on trials directly preceding errors (see Fig. 1, left panel). By only comparing trials that are proximal in time, this method controls for global fluctuations in task performance that confound more traditional measures of PES that compare posterror and postcorrect RTs (Dutilh et al., 2012), although it should be noted that this method does not take into account the effect of pre-error speedup (Dudschig & Jentzsch, 2009). Specifically, we isolated the error triplets from our data. Then, the mean RT on pre-error trials was subtracted from the mean RT on posterror trials for each participant, to obtain PES scores. We used repeated measures ANOVA with a 2 (Trial: pre-error or posterror trial) × 2 (Task: flanker or switch) within-subjects design to infer whether the participants showed PES.

We also assessed whether participants showed an increase in posterror accuracy (PEA) by comparing the accuracy on trials directly following errors with the accuracy on trials directly following correct responses. For this calculation, first the mean accuracy per person on trials subsequent to correct responses and trials subsequent to errors was computed. The error and correct trials only included incongruent trials for the flanker task and switch trials for the switch task. Then, the mean accuracy on the postcorrect trials was subtracted from the mean accuracy on the posterror trials for each participant to obtain the increase in PEA scores. A repeated measures ANOVA with a 2 (Trial: postcorrect or posterror) × 2 (Task: flanker or switch) within-subjects design was employed to infer whether participants showed an increase in PEA.

### Cardiac analyses

The preprocessed IBI and RZI signals were imported in Brain Vision Analyzer. Per participant and per task, the IBI and RZI data were segmented into error triplets and correct triplets. Next, the waveforms were further segmented in separate pre-error, error, and posterror segments, and precorrect, correct, and postcorrect segments, with Time Point 0 reflecting stimulus presentation. The intervals ranged from stimulus onset to 4,000 ms after stimulus onset. A baseline correction was performed on these segments so that the mean IBI and RZI during a period of – 2 to – 1 s relative to pre-error and precorrect trial onset was subtracted from these segments. Thus, this baseline was subtracted from the pre-error/precorrect, error/correct, and posterror/postcorrect segments. Note that although frequently a baseline period closer to stimulus onset is chosen (e.g., – 1 s to stimulus onset), we chose this particular baseline in order to avoid contamination by interpolated values from heart rate changes immediately following stimulus onset (see Kuipers et al., 2017). Finally, these six segments were each averaged per participant, so that each participant had an average waveform of each of the six segments per task. Whereas the main analyses in this article focus on the waveforms averaged across tasks (flanker and switch), we also report supplementary analyses on the data for both tasks separately, for reasons of completeness.

Using these averaged waveforms, we established whether there was an increased orienting response and/or an increase in effort mobilization in response to errors and whether this reaction occurred during error trials or posterror trials. We statistically compared waveforms using paired *t* tests that were corrected for multiple comparisons using the BESA Statistics nonparametric permutation cluster analysis tool, based on 10,000 permutations and an initial cluster threshold of *p* < .05 (Maris & Oostenveld, 2007). Using this method, we performed paired *t* tests in which we compared pre-error with precorrect, error with correct, and posterror with postcorrect waveforms, for both the IBI and RZI measures and across tasks. These comparisons are depicted in Fig. 1 (middle panel). We also tested whether these differences in waveforms observed in the current trials (error, correct) and post trials (posterror, postcorrect) differed in magnitude when subtracted from the difference in pre trials (pre-error, precorrect). For reasons of completeness, we also compared the waveforms between tasks, by testing whether the differences in waveforms observed in the pre trials (pre-error, precorrect), current trials (error, correct), and post trials (posterror, postcorrect) differed between tasks, with the analyses for each task reported separately. For all waveforms, statistical inferences were based on clusters with a cluster-corrected *p* < .05 (Maris & Oostenveld, 2007).

### Multilevel analyses

Next, we investigated whether changes in IBI and RZI in the error-trial and posterror-trial waveforms could predict PES. We employed multilevel regression models (implemented in R version 3.3.1, package lme4; Bates, Mächler, Bolker, & Walker, 2015), because these take into account the dependencies (correlations) between the scores of the same participant that occur because of the hierarchical structure of the data. Moreover, as such, we allowed for individual differences in the relationship between IBI or RZI and posterror RT. In our case, the multilevel structure was defined by trials (Level 1) nested in subjects (Level 2). Three models were fitted. Model 1 focused on the relationship between the mean IBI during error trials and posterror RT. Model 2a focused on the relationship between the mean RZI during posterror trials and posterror RT. Model 2b was added as a control analysis and focused on the relationship between the mean RZI during postcorrect trials and postcorrect RT. This control model allowed us to exclude the possibility that the effects observed in Model 2a reflected a general effect (rather than an error-specific effect) that was also visible in correctly performed trials. A summary of the used models is shown in Fig. 1, right panel.

For all models, parameters were estimated using the full maximum likelihood procedure. Models were fitted in a bottom-up fashion following the recommendations by Hox (2010). That is, analogue to step-wise regression in the multiple regression domain, we started with a simple model and added parameters if their inclusion improved model fit. We started with an unconditional means (intercept-only) model. Then, the Level 1 predictors were added as fixed effects only. Next, the Level 1 predictors were included as random effects when their inclusion improved the model fit. Finally, we explored whether the inclusion of interaction effects between the Level 1 predictors (two-way, three-way, and four-way interactions) would significantly improve the model fit. In order not to make the model too complex, which might yield estimation problems, we only added these interactions between Level 1 predictors as fixed effects. Likelihood ratio tests were used to establish whether the inclusion of random effects and interaction effects would significantly improve model fit.

Assumptions of normality and homoscedasticity were checked by means of inspection of the residuals of the models. Because of the observation of some deviations from normality in the residuals, we additionally performed linear regressions with a clustered bootstrap procedure to further establish the significance of the predictors (Davison & Hinkley, 1997). Bootstrapping does not require distributional assumptions and can therefore provide valid inferences when assumptions of the multilevel model are violated. A clustered bootstrap procedure with 10,000 bootstrap samples was employed and parametric 95% confidence intervals (CIs) were calculated for the parameter estimates (regarding fixed effects). We also tested the final models of Model 1 and Model 2a using a more robust multilevel method (package robustlmm; Koller, 2016) that produced the same significant effects.

For these analyses, all error and correct triplets were isolated from the physiological IBI and RZI data. Then, single-trial IBI and RZI predictors for error (Model 1), posterror (Model 2a), and postcorrect trials (Model 2b) were extracted using the mean interval from 1 to 4 s following stimulus onset of the respective trials. Baseline correction was performed for each segment, such that the mean IBI and RZI across a period of – 2 to – 1 s relative to the pre-error (Model 1 and 2a) and precorrect (Model 2b) stimulus presentations were subtracted from these values. We will use the terms *error IBI* and *error RZI* when referring to analyses on the waveforms locked to stimulus onset of the error trials, whereas we will adopt the terms *posterror IBI* and *posterror RZI* when referring to analyses on the waveforms locked to stimulus onset of the posterror trials, and the terms *postcorrect IBI* and *postcorrect RZI* when referring to analyses on the waveforms locked to stimulus onset of the postcorrect trials.

### Detailed description of the predictors used in the multilevel models

Model 1 focused on the relationship between the heart rate orienting response and PES. The predictor of main interest was error IBI and other predictors consisted of: pre-error RT (RT on the trial that preceded the error), error RZI, and task (flanker = 0, switch = 1). Error RZI was forced into the model to establish the unique contribution of both the error trial IBI and error trial RZI. In this model all the predictors were included as fixed effects. Furthermore, pre-error RT and task were added as random effects as their inclusion significantly improved model fit. Also, error IBI was included as random effect because this variable concerned the variable of main interest and individual differences in the effect of this variable were expected. Note that the exclusion of this variable as random effect did not notably change parameter estimates or significance levels. Finally, model fit was improved with the inclusion of a (fixed) interaction between pre-error RT and error IBI.

Model 2a focused on the relationship between effort mobilization and PES. The predictor of main interest was posterror RZI and other predictors consisted of: pre-error RT, posterror IBI, and task (flanker = 0, switch = 1). Posterror IBI was forced into the model to establish the unique contributions of both posterror RZI and posterror IBI. All predictors were included as fixed effects and pre-error RT and task were added as random effects as their inclusion significantly improved the model fit. Posterror RZI was also included as a random effect, because this variable concerned the variable of main interest and individual differences in the effect of this variable were expected. Note that the exclusion of this variable as a random effect did not notably change the parameter estimates or significance levels. Finally, model fit was improved with the inclusion of a (fixed) interaction between task and posterror IBI.

Model 2b was fitted in order to demonstrate that the effect observed in Model 2a could not be attributed to a general effect of RZI on RT also visible in correctly performed trials. The predictor of main interest was postcorrect RZI and other predictors consisted of precorrect RT (RT on the trial that preceded the correct trial), postcorrect IBI, and task (flanker = 0, switch = 1). Postcorrect IBI was forced into the model to establish the unique contribution of both the postcorrect RZI and postcorrect IBI. All predictors were included as fixed effects, and precorrect RT and task were added as random effects because their inclusion significantly improved the model fit. Postcorrect RZI was also included as a random effect, analogously to Model 2a. The model fit was further improved with the inclusion of (fixed) interactions between task and postcorrect IBI, task and precorrect RT, postcorrect RZI and precorrect RT, and postcorrect RZI and postcorrect IBI.

In all models the RT variables (pre- and posterror RT, pre- and postcorrect RT) represented log-transformed RTs in order to approach a normal distribution for these variables. Also, *z*-scoring of the predictor variables (except task, which is dichotomous) was carried out on a within-subjects basis (i.e., using participant-specific across-trial mean and *SD* values).

## Results

### Behavioral results

We first established whether the congruency effect and switch effect were present in the flanker and switch task, respectively. Participants indeed showed the expected congruency effect in the flanker task [*t*(46) = 16.51, *p* < .001], indicating that participants were slower on incongruent trials (*M* = 505.88 ms, *SD* = 50.28) than on congruent trials (*M* = 466.40 ms, *SD* = 52.36). Additionally, participants were less accurate on incongruent (*M* = 90.70%, *SD* = 4.28) than on congruent trials (*M* = 96.87%, *SD* = 2.32) [ *t*(46) = – 13.73, *p* < .001]. We also observed the expected switch effect in the switch task [*t*(46) = 8.17, *p* < .001], indicating that participants were slower on trials in which they had to switch tasks (*M* = 827.97 ms, *SD* = 188.24) than on trials in which they had to repeat the same task as in the previous trial (*M* = 753.19 ms, *SD* = 152.12); note that participants were not significantly less accurate on switch trials (*M* = 92.49%, *SD* = 0.037) than on repetition trials (*M* = 93.15%, *SD* = 0.039) [*t*(46) = – 1.75, *p* = .087].

Second, we used repeated measures ANOVA to test whether there were differences in the RTs between error and correct trials (Table 1). Overall, RTs were longer in the switch task than in the flanker task [*F*(1, 46) = 212.77, *p* < .001, *MSE* = 30,651.83, *η*_p_^2^ = .82]. Moreover, RTs were shorter on error trials than on correct trials [*F*(1, 46) = 25.62, *p* < .001, *MSE* = 3,707.68, *η*_p_^2^ = .36], but this effect depended on task [*F*(1, 46) = 7.00, *p* = .011, *MSE* = 3,244.01, *η*_p_^2^ = .13]. Post-hoc paired *t* tests revealed that RTs were significantly shorter on error than on correct trials for the flanker task [*t*(46) = – 12.46, *p* < .001], but not for the switch task [*t*(46) = – 1.41, *p* = .166].

Finally, we used repeated measures ANOVA to compare posterror trials with pre-error trials regarding both RTs and accuracy, which established the presence of a posterror slowing effect [*F*(1, 46) = 11.97, *p* = .001, *MSE* = 3,250.05, *η*_p_^2^ = .206] and an increase in posterror accuracy [*F*(1, 46) = 7.56, *p* = .008, *MSE* = 0.001, *η*_p_^2^ = .141] (see Table 1). In addition, overall, participants responded faster in the flanker task than in the switch task [*F*(1, 46) = 189.01, *p* < .001, *MSE* = 23,899.3, *η*_p_^2^ = .804]. No other significant effects or interactions were observed.

### Cardiac results

We subsequently tested whether errors resulted in cardiac changes in IBI and RZI during the waveforms for error versus correct trials and for posterror versus postcorrect trials. Figure 1 (middle panel) summarizes the main comparisons and the results of this analysis.

**Fig. 2:**
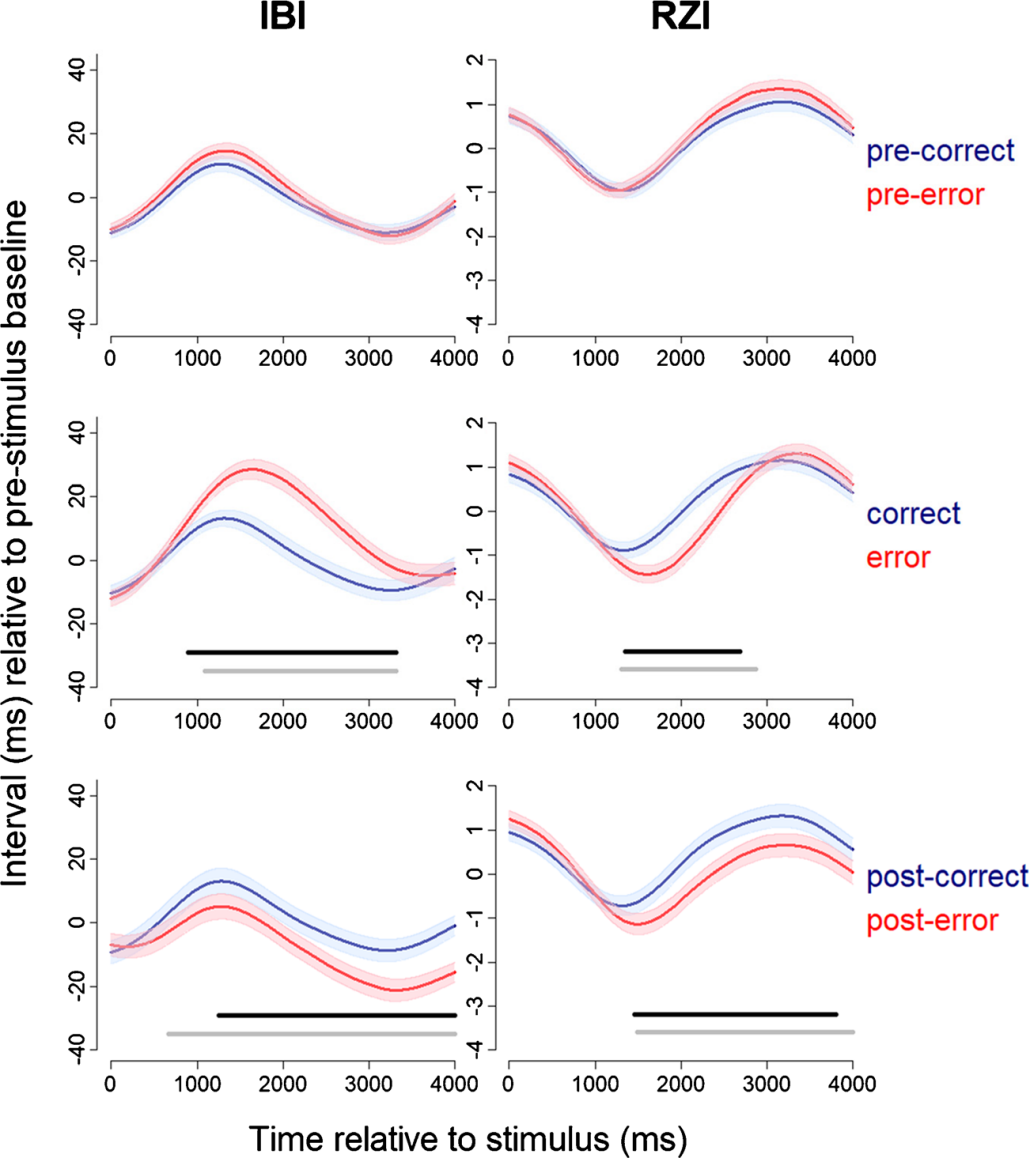
Effect of errors on interbeat intervals (IBI; left panels) and RZ intervals (RZI; right panels), during pre-error trials (upper panels), error trials (middle panels), and posterror trials (lower panels). Time point 0 depicts stimulus onset. Standard errors are plotted around the waveforms. Black lines indicate significant clusters (corrected *p* < .05) when comparing the waveforms (error and correct, posterror and postcorrect), and gray lines indicate significant clusters when comparing the difference wave for either error versus correct or posterror versus postcorrect with the difference wave for pre-error versus precorrect.

Figure 2 shows the IBI and RZI waveforms for pre-error and precorrect trials, error and correct trials, and posterror and postcorrect trials. Across trial types, the IBI waveforms showed the typical pattern of initial anticipatory heart rate deceleration preceding the response, which was followed by acceleratory recovery (Jennings & van der Molen, 2002, 2005; Jennings, van der Molen, Brock, & Somsen, 1991; Somsen, Jennings, & van der Molen, 2004). Cardiac deceleration was, however, significantly more pronounced during errors than during correct trials (cluster statistic = 11,246.1, mean IBI error = 16.92, mean IBI correct = 2.34, corrected *p* < .001, time interval = 893–3,319 ms). It is unlikely that this effect can be attributed to differences in acceleratory recovery after the response, because the difference between RTs on error and correct trials was very small (66.93 ms for the flanker task and 22.99 ms for the switch task; see Table 1). Thus, cardiac deceleration was increased during error trials, which can be interpreted as an orienting response. Notably, during posterror trials a significant decrease in IBI was observed (cluster statistic = – 8,163.3, mean IBI posterror = – 11.23, mean IBI postcorrect = – 0.83, corrected *p* < .001, time interval = 1,258–4,000 ms). The RZI decreased significantly during error trials as compared to correct trials (cluster statistic = – 5,162.2, mean RZI error = – 0.791, mean RZI correct = 0.002, corrected *p* < .001, time interval = 1,350–2,683). However, this decrease in RZI cannot be interpreted as an increase in effort mobilization, because it was accompanied by an increase in IBI (Frank–Starling effect; Obrist, 1981)—that is, the greater ventricular filling caused by the increase in IBI leads to stronger and faster contractions, and this shorter RZI is thus likely not driven by sympathetic influences (Sherwood et al., 1990). A significant decrease in RZI was also observed during posterror as compared to postcorrect trials (cluster statistic = – 6,465.8, mean RZI posterror = 0.030, mean RZI postcorrect = 0.724, corrected *p* = .0036, time interval = 1,462–3,804 ms). Since it was not accompanied by an increase in IBI, this decrease in RZI can be safely interpreted as an increase in cardiac effort. There was no significant difference between the IBI and RZI waveforms when comparing pre-error with precorrect trials.

Next, we tested whether these differences in waveforms observed in the current trials (error, correct) and post trials (posterror, postcorrect) differed from the difference in the pre trials (pre-error, precorrect). To this end, we compared the difference wave for error versus correct with the pre-error versus precorrect wave, and the difference wave for posterror versus postcorrect with the pre-error versus precorrect wave (gray lines in Fig. 2). The magnitude of the difference wave was larger for the current trials than for the pre trials for both IBI (cluster statistic = 9,258.2, mean difference error – correct = 15.37, mean difference pre-error – precorrect = 2.18, corrected *p* < .001, time interval = 1,093–3,313) and RZI (cluster statistic = – 7,240.8, mean difference error – correct = – 0.719, mean difference pre-error – precorrect = 0.188, corrected *p* < .001, time interval = 1,303–2,866). Also, the magnitude of the difference wave was larger for the post trials than for the pre trials for both IBI (cluster statistic = – 10,226.3, mean difference posterror – postcorrect = – 9.84, mean difference pre-error – precorrect = 1.83, corrected *p* < .001, time interval = 664–4,000) and RZI (cluster statistic = – 7,786.6, mean difference posterror – postcorrect = – 0.683, mean difference pre-error – precorrect = 0.233, corrected *p* = .0025, time interval = 1,492–4,000). These results confirm that the changes in IBI and RZI observed in the current and post trials were significantly larger than those in the pre trials.

Additionally, for reasons of completeness, we compared the difference waves of the pre trials, current trials, and post trials between tasks. We only observed a significant difference between the flanker and switch tasks in the error versus correct difference waveforms (gray lines in Figs. S1 and S2). That is, the magnitude of the difference between the error and correct waveforms was larger in the switch task than in the flanker task for both IBI (cluster statistic = – 4,968.8, mean difference error – correct flanker = 4.196, mean difference error – correct switch = 18.953, corrected *p* = .0092, time interval = 2,092–3,800) and RZI (cluster statistic = 3741.4, mean difference error – correct flanker = 0.075, mean difference error – correct switch = – 1.197, corrected *p* = .017, time interval = 1,890–3,172). Thus, the increase in IBI and the decrease in RZI in response to errors was larger in the switch task than in the flanker task. We also ran the analyses in which we compared pre-error with precorrect, error with correct, and posterror with postcorrect waveforms, for both tasks separately. The results of these analyses are shown in Fig. S1 for the IBI and Fig. S2 for the RZI.

### Multilevel analyses predicting influence of cardiac measures on behavior

Next, we used the error IBI waveform as an indication of orienting response and the posterror RZI waveform as an indication of effort mobilization and investigated, in two separate multilevel models, whether these variables predicted posterror RTs. In a third model, we additionally tested whether postcorrect RZI predicted postcorrect RTs, to control for the possibility of a general effect of RZI on RT visible in correctly performed trials. A summary of these models and of the main findings is shown in Fig. 1 (right panel).

Model 1 investigated whether the IBI waveform in error trials predicted posterror RTs. The results of this model are presented in Table 2. As expected, this model showed that the pre-error RT was positively related to the posterror RT and that posterror RTs were larger in the switch task than in the flanker task. Also, this model suggested that error IBI predicted the posterror RT at a marginally significant level; the significance of this effect was further supported by results from the clustered bootstrap procedure. The direction of this effect was, however, opposite to what was expected. That is, an increase in IBI during error trials was related to a decrease as opposed to an increase in posterror RT. Therefore, our analyses did not confirm a positive relation between the cardiac orienting response and posterror RT slowing. The interaction effect included in the model showed that the relationship between error IBI and posterror RT was influenced by the pre-error RT, but the direction of the effect remained the same, since an increase in pre-error RT was related to an even stronger negative relation between error IBI and the posterror RT. We found no relationship between error RZI and the posterror RT.

**Table 2:** Parameter estimates of the multilevel model investigating the relation between error IBI and posterror RT

Fixed Effects	Estimate	*SE*	95% CI	*t* Value (df)	*p* Value	Bootstrapped 95% CI	Random Effects	
Intercept	6.21	.019	[6.17, 6.25]	329.48 (45.3)	<.001	[6.15, 6.23]	${\sigma}_e^2$ Residual	.053
Error IBI	– .010	.006	[– .022, .0008]	– 1.82 (134.4)	.071	[– .022, – .001]	${\sigma}_0^2$ Intercept	.014
Error RZI	– .003	.005	[– .014, .007]	– 0.58 (1974)	.566	[– .011, .008]	${\sigma}_1^2$ Error IBI	.0002
Pre-error RT	.038	.008	[.022, .054]	4.67 (33.9)	<.001	[.013, .051]	${\sigma}_2^2$ Pre-error RT	.0008
Task	.387	.022	[.344, .430]	17.70 (39.4)	<.001	[.346, .462]	${\sigma}_3^2$ Task	.012
Error IBI * Pre-error RT	– .015	.005	[– .026, – .005]	– 2.87 (1527)	.004	[– .030, – .002]		

IBI = interbeat interval, RT = reaction time, RZI = RZ interval

Model 2a investigated whether the RZI waveform in posterror trials predicted the posterror RT. The results of this model are presented in Table 3. As in the previous model, the pre-error RT and task significantly predicted the posterror RT. Furthermore, this model revealed a significant negative relationship between posterror RZI and the posterror RT. Thus, in line with the cognitive-control account of posterror slowing, an increase in effort mobilization, as reflected by a smaller mean RZI during posterror trials, was related to a larger posterror RT. There was no main effect of posterror IBI on the posterror RT. The model did, however, show a significant interaction effect between task and posterror IBI, indicative of a positive relationship between posterror IBI and the posterror RT in the switch task. However, this effect should be interpreted with some caution, because (the significance of) this effect was not supported by results from the clustered bootstrap procedure (i.e., its significance depended on the tenability of the assumptions of the multilevel model).

**Table 3:** Parameter estimates of the multilevel model investigating the relation between posterror RZI and posterror RT

Fixed Effects	Estimate	*SE*	95% CI	*t* Value (df)	*p* Value	Bootstrapped 95% CI	Random Effects	
Intercept	6.21	.019	[6.17, 6.25]	330.41 (45.3)	<.001	[6.15, 6.23]	${\sigma}_e^2$ Residual	.053
Posterror IBI	– .0005	.007	[– .013, .012]	– 0.08 (1958)	.934	[– .007, .013]	${\sigma}_0^2$ Intercept	.014
Posterror RZI	– .015	.005	[– .025, – .004]	– 2.64 (154.1)	.009	[– .021, – .002]	${\sigma}_1^2$ Posterror RZI	.0001
Pre-error RT	.038	.008	[.022, .054]	4.54 (34.9)	<.001	[.013, .050]	${\sigma}_2^2$ Pre-error RT	.001
Task	.383	.022	[.340, .426]	17.38 (38.9)	<.001	[.343, .460]	${\sigma}_3^2$ Task	.012
Task * Posterror IBI	.023	.011	[.002, .045]	2.15 (1973)	.032	[– .019, .039]		

IBI = interbeat interval, RT = reaction time, RZI = RZ interval

Model 2b investigated whether the RZI waveform in postcorrect trials predicted the postcorrect RT. This control model was run to exclude the possibility that a general effect of RZI on RTs could explain the findings in Model 2a. The results of this model are presented in Table 4. Main effects of both precorrect RT and task were present, with these variables positively predicting postcorrect RTs. Importantly, no significant effect was found of postcorrect RZI on postcorrect RTs, which confirms that only after error commission was increased effort mobilization related to RT. In addition, postcorrect IBI positively predicted postcorrect RTs, which might reflect a combination of sustained cardiac deceleration and/or delayed cardiac acceleration on trials in which the responses were slow. Additionally, this model showed an interaction effect between task and postcorrect IBI, which indicates a stronger positive relationship between IBI and RT in the switch task. Also, a positive interaction between task and precorrect RT, a positive interaction between precorrect RT and postcorrect RZI, and a negative interaction between postcorrect RZI and postcorrect IBI were found, although these effects were not supported by the clustered bootstrap procedure, and thus should be interpreted with care.

**Table 4:** Parameter estimates of the multilevel model investigating the relation between postcorrect RZI and postcorrect RT

Fixed Effects	Estimate	*SE*	95% CI	*t* Value (df)	*p* Value	Bootstrapped 95% CI	Random Effects	
Intercept	6.184	.015	[6.179, 6.188]	426.45 (47.0)	<.001	[6.156, 6.210]	${\sigma}_e^2$ Residual	.063
Postcorrect IBI	.0054	.002	[.0047, .0061]	2.40 (20620)	.016	[.0017, .0100]	${\sigma}_0^2$ Intercept	.010
Postcorrect RZI	– .0025	.002	[– .0030, – .0019]	– 1.35 (45)	.185	[– .006, .001]	${\sigma}_1^2$ Postcorrect RZI	.00002
Precorrect RT	.0233	.004	[.0222, .0244]	5.94 (63)	<.001	[.017, .028]	${\sigma}_2^2$ Precorrect RT	.0005
Task	.4179	.019	[.4124, .4234]	21.53 (47)	<.001	[.384, .461]	${\sigma}_3^2$ Task	.017
Postcorrect RZI * Postcorrect IBI	– .0048	.001	[– .0052, – .0044]	– 3.46 (20840)	<.001	[– .0076, .0012]		
Postcorrect RZI * Precorrect RT	.0055	.002	[.0050, .0059]	3.35 (21790)	<.001	[– .0018, .0058]		
Task * Postcorrect IBI	.0107	.003	[.0097, .0116]	3.16 (22490)	.002	[.0025, .0185]		
Task * Precorrect RT	.0092	.003	[.0082, .0101]	2.72 (22500)	.007	[– .002, .021]		

IBI = interbeat interval, RT = reaction time, RZI = RZ interval

## Discussion

The present findings provide novel support for the notion that posterror slowing (PES) is related to increased effort mobilization, which is in line with a cognitive-control account of PES (Botvinick et al., 2001). Specifically, our results showed a decrease in RZI during posterror trials, and more importantly, this decrease predicted adaptive changes in PES at the individual level. These findings suggest that errors increase the adaptive recruitment of effort in trials subsequent to an error. Notably, the decrease in RZI during posterror trials was accompanied by an increase in heart rate. The direction of these effects indicate that the shortened RZI reflects cardiac effort and cannot be attributed to physiological confounds (Obrist, 1981). Interestingly, the increase in heart rate itself was not related to PES, which is in line with earlier work that has defined effort in terms of myocardial sympathetic activity, which is not possible to measure using simple measures of heart rate (Gendolla et al., 2011).

Replicating earlier studies (Crone et al., 2003; Danev & Winter, 1971; Fiehler et al., 2004; Hajcak et al., 2003; Somsen et al., 2000; van der Veen et al., 2000; Wessel et al., 2011), we additionally observed heart rate slowing in response to errors, suggesting that errors also evoke an orienting response (Notebaert et al., 2009; Notebaert & Verguts, 2011). According to the account by Notebaert et al. (2009) orienting reflects attention directed away from the task toward the error, which causes a delay in RT on the subsequent trial. However, other work has suggested that cardiac orienting might reflect inhibitory processes that actually help to suppress irrelevant information and facilitate task relevant processing (Jennings, 1992; van der Molen, 2000; but see also Crone et al., 2003). Relatedly, Murphy et al. (2016) proposed that the pupillary orienting response reflects adaptive control. Their findings indeed showed that pupil dilation to errors positively predicted PES and posterror accuracy. In contrast, our findings do not provide clear evidence for a relationship between cardiac deceleration and posterror slowing: If anything, cardiac deceleration during error trials tended to predict reduced PES. These results are hard to reconcile with an orienting account of PES. They also suggest that an interpretation in terms of effort might provide a more parsimonious account of earlier studies observing error-related pupil dilation. Indeed, traditionally pupil dilation has been interpreted in terms of effort mobilization (Kahneman, 1973; van der Wel & van Steenbergen, 2018). Alternatively, it is possible that different physiological indices tap different aspects of the orienting response, and that the latter should not be considered a unitary construct (Barry, 2006; Nieuwenhuis, De Geus, & Aston-Jones, 2011).

The results of the present study dovetail with recent neuroscientific work that has started to highlight the commonalities between effort and cognitive control. For example, adaptive increases in both cognitive control and effort are likely driven by the dorsal anterior cingulate cortex (dACC), a region that not only signals the occurrence of conflict (Botvinick et al., 2001; Botvinick, Cohen, & Carter, 2004), but also integrates information concerning the payoff and costs associated with exerting effort (Shenhav, Botvinick, & Cohen, 2013). Consistent with these accounts, numerous studies have implied the ACC in error related adjustments in cognitive control. For example, EEG studies have measured the so-called error-related negativity (Carter et al., 1998; Falkenstein, Hohnsbein, Hoormann, & Blanke, 1991; Gehring, Goss, Coles, Meyer, & Donchin, 1993), which has been related to PES in some studies (Debener et al., 2005; Fischer, Danielmeier, Villringer, Klein, & Ullsperger, 2016; Gehring et al., 1993). Additionally, fMRI studies have also revealed increased activation in the ACC in response to errors as a predictor of PES (Braver, Barch, Gray, Molfese, & Snyder, 2001; Carter et al., 1998; Klein et al., 2007). It is likely that the dACC is also the source of cardiovascular effort mobilization, as it has been associated with the sympathetic modulation of heart rate (Critchley et al., 2003; Silvestrini, 2017). In addition, activity in the dorsolateral prefrontal cortex in response to errors (Garavan, Ross, Murphy, Roche, & Stein, 2002) and in the ventrolateral prefrontal cortex during posterror trials (Li et al., 2008) has been associated with PES. Thus, the dACC is part of a larger cognitive-control network, and interactions between dACC and more lateral prefrontal areas support the adaptive changes in behavior and cardiac effort following errors.

This study has some limitations that must be acknowledged. First, we were unable to infer with certainty when exactly the error-evoked increase in cardiac effort started to occur. Although we have emphasized the RZI effect during the posterror trial, a decrease in RZI also occurred earlier in the error trial itself. However, the RZI effect in the error trial itself was likely driven by the concomitant increase in IBI (Frank–Starling effect; Obrist, 1981), so it is problematic to attribute this to cardiac effort (cf. Kuipers et al., 2017). Second, it is unclear whether the increase in effort mobilization was evoked by the error commission or by the posterror trial itself. Although we observed an increase in effort mobilization following posterror stimulus presentation approximately 1.5 s following stimulus onset, it is possible that this reaction is relatively slow and actually reflects a late response to the error commission in the current trial. Because the intervals between trials had a limited jitter and were not very long, we cannot rule out this possibility. However, it might be difficult to circumvent this issue in future studies, because error-related behavioral changes tend to diminish with long intervals (Jentzsch & Dudschig, 2009). Third, PES was assessed by comparing RTs on posterror trials with RTs on pre-error trials. Although this method circumvents the confound of global fluctuations in traditional measures of PES (Dutilh et al., 2012), it can overestimate PES, due to the effect of pre-error speedup (Dudschig & Jentzsch, 2009).

### Conclusions

The present study has revealed both orienting-related cardiac deceleration during error trials and increased cardiac effort mobilization during posterror trials. We found no evidence that cardiac orienting was related to PES. In contrast, we did find an association between effort mobilization and PES: Increases in cardiac effort, as reflected by a shorter RZI, were related to an increase in PES, in line with a cognitive-control account of PES. Our findings show that adaptive changes in behavior accompany a response in cardiac effort, rendering this a promising approach to be applied in future studies of cognitive effort.

#### Author note

We thank Ted Adrichem, Maxim Allaart, Esther Bliek, Anne de Groot, Anke Halfweeg, Jasmijn Hondebrink, Sanne de Jager, Mithras Kuipers, Tyrza van Leeuwen, Romy Petitjan, Alexander Skowron, Maria Tarisa, and Pauline van der Wel for their help in data acquisition and data preprocessing. We are grateful for helpful discussions with Sander Nieuwenhuis and Michael Richter.

## Electronic supplementary material

ESM 1 (DOCX 5501 kb)
