# Supplementary material for: Heart work after errors: Behavioral adjustment following error commission involves cardiac effort
Source: Cogn Affect Behav Neurosci. 2018 Feb 20;18(2):375–88. doi: 10.3758/s13415-018-0576-6 (PMC5889424; doi:10.3758/s13415-018-0576-6)
Supplement: Supplementary file 1 — (DOCX 5501 kb) [file 13415_2018_576_MOESM1_ESM.docx]

# Supplemental material

# Figure S1.


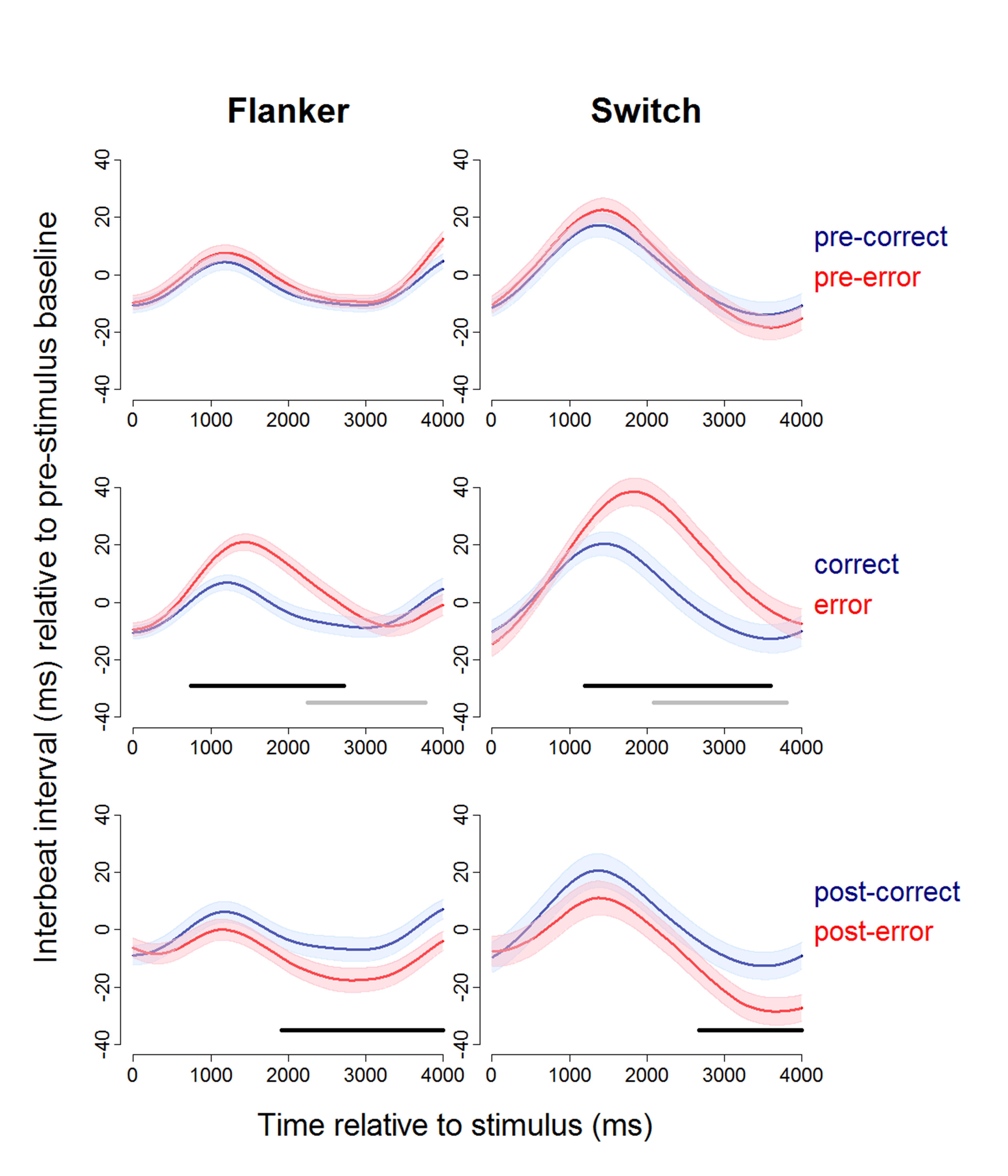


**Figure S1.** Effect of errors on inter beat interval for the flanker task (left panels) and switch task (right panels), during pre-error trials (upper panels), error trials (middle panels), and post-error trials (lower panels). Timepoint 0 depicts stimulus onset. Standard errors are plotted around waveforms. Black lines indicate significant clusters (corrected p < .05) when comparing waveforms (error and correct, post-error and post-correct). Gray lines indicate significant clusters when comparing the difference wave of the flanker task with the difference wave of the switch task. When comparing waveforms between tasks only significant differences were observed in the error vs correct waveforms.

# Figure S2.

#
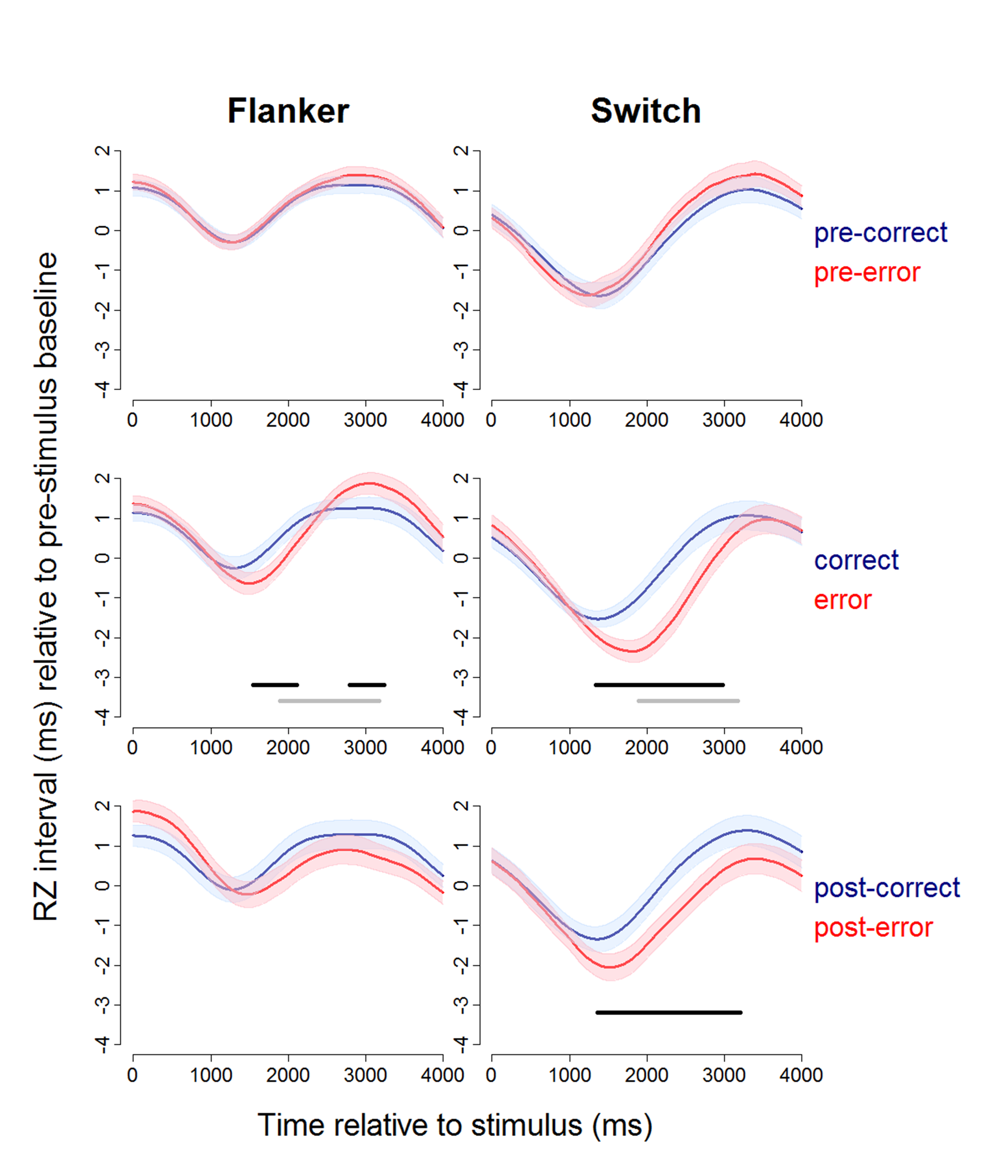


**Figure S2.** Effect of errors on RZ interval for the flanker task (left panels) and switch task (right panels), during pre-error trials (upper panels), error trials (middle panels), and post-error trials (lower panels). Timepoint 0 depicts stimulus onset. Standard errors are plotted around waveforms. Black lines indicate significant clusters (corrected p < .05) when comparing waveforms (error and correct, post-error and post-correct). Gray lines indicate significant clusters when comparing the difference wave of the flanker task with the difference wave of the switch task. When comparing waveforms between tasks only significant differences were observed in the error vs correct waveforms.
